# P-786. Rates of Treatment Discontinuation over Time with Rifampin versus Isoniazid for Latent Tuberculosis Infection: A 6-year Experience at a Large Safety-Net Clinic

**DOI:** 10.1093/ofid/ofae631.980

**Published:** 2025-01-29

**Authors:** Maria F Martins, Juan M Teran Plasencia, Pranay Sinha, Carlos Acuña-Villaorduña

**Affiliations:** Boston University Medical Center, Boston, Massachusetts; University of Nebraska Medical Center, Omaha, Nebraska; Boston University, Boston, Massachusetts; Boston University School of Medicine, Boston, Massachusetts

## Abstract

**Background:**

Treatment of latent tuberculosis infection (LTBI) is a crucial component of tuberculosis (TB) eradication plans in the United States (US). It is estimated that 80% of active TB cases arise from LTBI reactivation. Numerous gaps have been identified in the LTBI care cascade, leading to low rates of treatment initiation and completion. Treatment duration has shown to be a significant factor influencing completion rates for LTBI treatment. Since 2020, the Center for Disease Control and Prevention (CDC) recommends 4 months of daily rifampin (4R) as the preferred regimen for LTBI, while 6 to 9 months of isoniazid (9H) is now considered an alternative regimen.

**Table 1.**
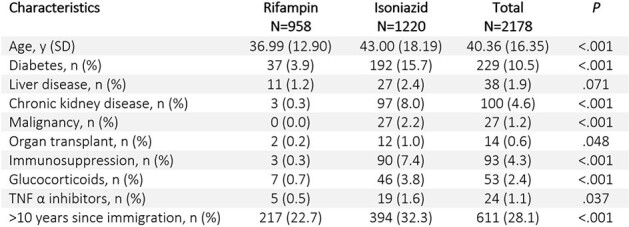
Characteristics of the Patients by Drug Used

**Methods:**

We conducted a retrospective cohort analysis including all patients treated for LTBI with 4R or 9H (May 2015 - September 2021) at the Boston Medical Center/Boston Public Health Commission TB Clinic. The primary objective was to compare completion rates between patients with LTBI treated with 4R versus 9H. For this, completion of 95% of the planned course was considered sufficient. We also compared rates of treatment discontinuation by plotting a survival curve. Data analysis was performed with *R* statistical software (version 4.3.2). The study was approved by the Institutional Review Board (IRB) of Boston University.

**Figure 1.**
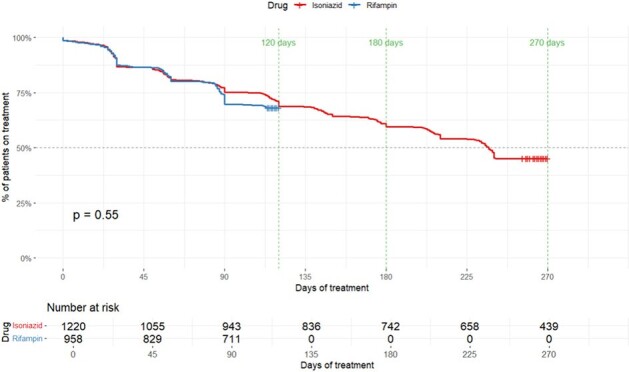
Rates of Treatment Discontinuation over Time for Rifampin versus Isoniazid

**Results:**

A total of 2178 patients with a median age of 40 years-old were treated for LTBI during the study period, 958 (43.99%) with 4R and 1220 (56.01%) with 9H (Table 1). The completion rates differed significantly, with 652 (68.1%) completing 4R compared to 550 (45.1%) completing 9H (p < 0.001). A Kaplan-Meier curve showed similar rates of discontinuation over time between the 2 regimens (Figure 1). After adjusting for key confounders using multivariable logistic regression model, the sole variable significantly associated with LTBI treatment completion was the use of 4R with an odds ratio of 2.805 (95% CI: 2.313 - 3.406) (p < 0.001) (Table 2).

**Table 2.**
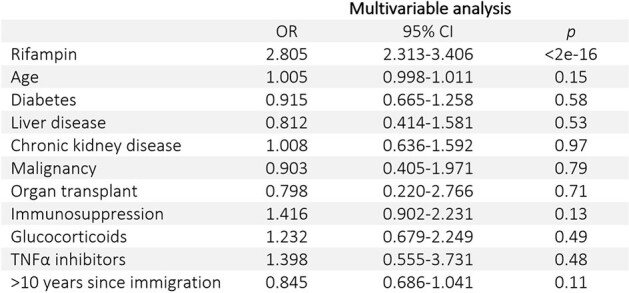
Factor's Association with Treatment Completion OR: odds ratio; CI: confidence interval

**Conclusion:**

Our findings confirm that 4R has better completion rates than 9H. Importantly, treatment completion on the survival curve was similar in the first 4 months of therapy, supporting the notion that treatment duration is a key factor determining LTBI treatment completion rate regardless of the drug used.

**Disclosures:**

**All Authors**: No reported disclosures

